# Estimating the Thumb Rotation Angle by Using a Tablet Device With a Posture Estimation Artificial Intelligence Model

**DOI:** 10.7759/cureus.59657

**Published:** 2024-05-04

**Authors:** Yutaka Ehara, Atsuyuki Inui, Yutaka Mifune, Hanako Nishimoto, Kohei Yamaura, Tatsuo Kato, Takahiro Furukawa, Shuya Tanaka, Masaya Kusunose, Shunsaku Takigami, Ryosuke Kuroda

**Affiliations:** 1 Department of Orthopaedic Surgery, Kobe University Graduate School of Medicine, Kobe, JPN

**Keywords:** thumb movement, range of motion (rom), tele medicine, machine learning (ml), artificial intelligence (ai)

## Abstract

MediaPipe Hand (MediaPipe) is an artificial intelligence (AI)-based pose estimation library. In this study, MediaPipe was combined with four machine learning (ML) models to estimate the rotation angle of the thumb. Videos of the right hands of 15 healthy volunteers were recorded and processed into 9000 images. The rotation angle of the thumb (defined as angle θ from the palmar plane, which is defined as 0°) was measured using an angle measuring device, expressed in a radian system. Angle θ was then estimated by the ML model by using parameters calculated from the hand coordinates detected by MediaPipe. The linear regression model showed a root mean square error (RMSE) of 12.23, a mean absolute error (MAE) of 9.9, and a correlation coefficient of 0.91. The ElasticNet model showed an RMSE of 12.23, an MAE of 9.95, and a correlation coefficient of 0.91; the support vector machine (SVM) model showed an RMSE of 4.7, an MAE of 2.5, and a correlation coefficient of 0.99. The LightGBM model achieved high values: an RMSE of 4.58, an MAE of 2.62, and a correlation coefficient of 0.99. Based on these findings, we concluded that the thumb rotation angle can be estimated with high accuracy by combining MediaPipe and ML.

## Introduction

Measuring the range of motion of the hand enables hand surgeons and therapists to assess hand function objectively and quantitatively [1-4]. An accurate measurement of the kinematics of the thumb can be achieved by using several well-known techniques, and optical systems featuring infrared cameras and reflective markers are considered the gold standard method [5]. Moreover, some studies have used a three-axis gyroscope [6]. Manual goniometers are still commonly used by clinicians. However, its measurement requires face-to-face evaluation and physical contact. This has become particularly challenging after the coronavirus disease 2019 (COVID-19) pandemic, thereby highlighting the importance of telemedicine for remote consultations and evaluations [4].

The use of smartphones for medical photography has become increasingly popular and accepted among a lot of patients, making smartphones an acceptable tool in telemedicine [7]. However, measuring complex finger joint movements via images and video is difficult [8-10]. Among finger movements, the thumb plays an important role in grasping and pinching movements. The thumb motion is defined as a combination of palmar abduction, radial abduction, flexion, and extension [11-13]. Various definitions of thumb motion have been reported in the literature; e.g., palmar abduction has been defined in the following ways: “the movement in which the thumb metacarpal moves away from the index metacarpal, perpendicular to the plane of the palm” or “the angle between the first and second metacarpals with the thumb maximally abducted” [14,15]. Previous research has reported the abduction movements of the thumb being simulated as the motion of a cone [16,17]. This method enables simultaneous evaluation of radial and palmar abduction, as well as evaluating radial and palmar abduction from the movement without the goniometers being applied during rehabilitation.

In 2020, Google introduced MediaPipe, which estimates and detects coordinates in real-time from images and videos of the whole body and hands [18,19]. By focusing on detecting the bounding box of a relatively rigid body part, the method uses a minimally sufficient number of key points on the face, hands, and feet to estimate the rotation, size, and position of the region of interest for the subsequent model. Although reports of the range of motion assessment for hands using MediaPipe have emerged, the evaluation of automated hand motion alone using MediaPipe with smartphone camera functionality lacks accuracy. When analyzing images of a specific protocol position captured with a smartphone, the average difference from the goniometer was −2.21 ± 9.29°[4].

According to one study, the integration of MediaPipe with artificial intelligence (AI) models for posture analysis can improve accuracy in assessing shoulder abduction angles [20]. In this study, the mean absolute percentage error (MAPE) was 1.539% to detect the abduction angle using MediaPipe and machine learning (ML). We hypothesized that integrating MediaPipe and ML could estimate the thumb rotation angle (defined as angle θ from the palmar plane, which is defined as 0°) with high accuracy. The purpose of this study was to develop a method to easily represent abduction motion and measure abduction angles in various movements of the thumb.

## Materials and methods

For representing the motion of the thumb in a simplified manner, the thumb rotation angle was approximated by a cone, assuming that the carpometacarpal (CM), metacarpophalangeal (MP), and interphalangeal (IP) joints are on the same straight line. An ML model was developed to estimate the thumb rotation angle (θ) using measurements from a protractor as the true values. Four ML models - linear regression, ElasticNet, support vector machine (SVM), and LightGBM - were employed to compare performance. The models were evaluated using the root mean square error (RMSE), the mean absolute error (MAE), and the correlation coefficient.

1. Participants

To evaluate the rotation angle of the thumb, 15 subjects (10 males and 5 females; mean age: 33.2± 7.39 years) were included. Participant inclusion criteria were as follows: both hands in good condition without any loss of fingers, no symptoms or abnormal findings on physical examination, and age above 18 years. Participants with any musculoskeletal disorder, pain, or deformity that influenced the hand movement and those who were unwilling to participate were excluded. Between April 2023 and December 2023, 15 Japanese volunteers who lived in Kobe were invited to participate in the study. Their age, gender, and dominant hand were recorded. The participants fully understood the study protocol and cooperated to perform the requested gestures. The study was approved by the Kobe University Ethics Committee (approval number: B210009), and informed consent was obtained from all participants.

**Figure 1:**
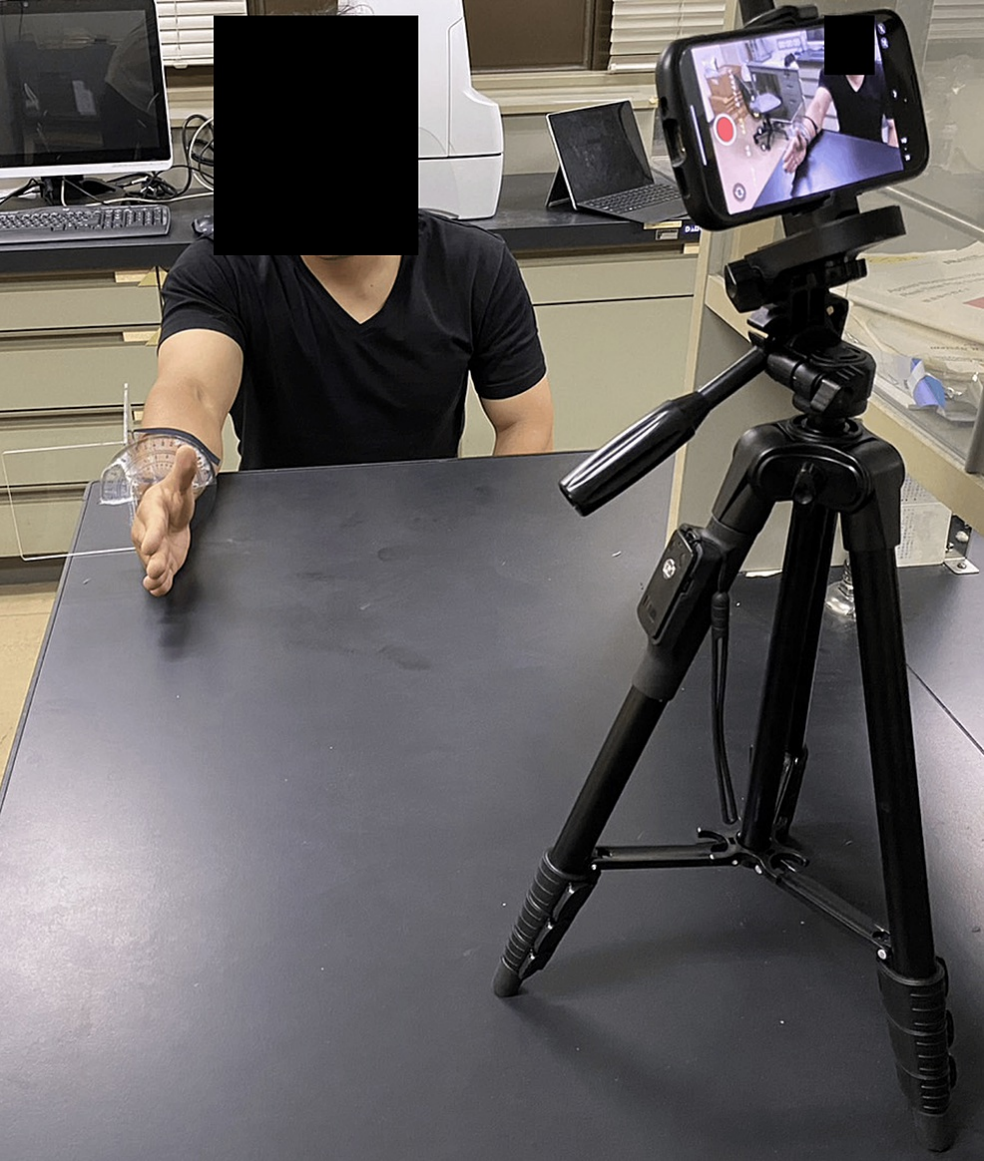
The camera recording position

A tablet device (iPhone 11Pro Max, Apple Inc., Cupertino, CA) was placed 50 cm above the table, 1 m on the palmar side, and at 45° from the subject’s right hand. The subjects were seated and in the mid-position of the wrist joint (Figure 1). All participants were right-handed. Participants were instructed to wear an angular device for measurement. They performed a rotation movement of the thumb.

2. Measurements

**Figure 2:**
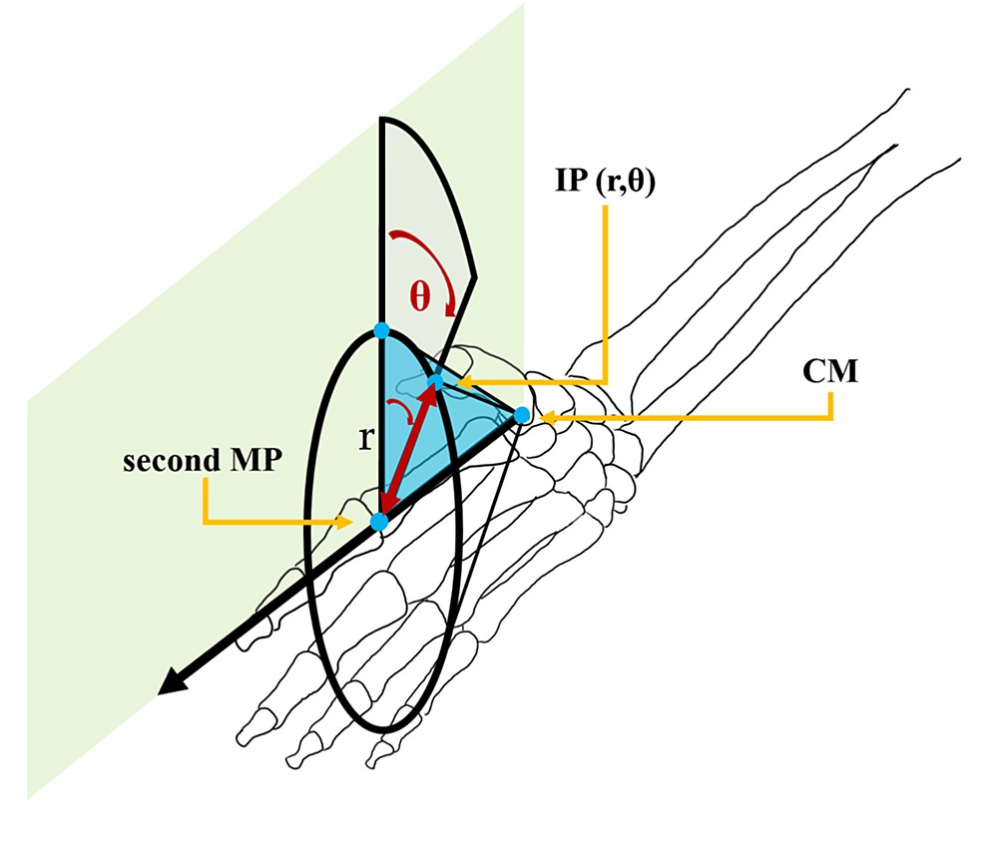
The definition of the coordinate system The abduction motion of the thumb was defined as a planar rotational motion of the thumb interphalangeal (IP) joint, with the second metacarpophalangeal (MP) joint as the center of rotation and the second metacarpal as the base axis. The position of the IP joint of the thumb is described as rotational displacement coordinates (r, θ) based on the distance r from the base axis and the rotation angle θ from the palmar plane, which is defined as 0° Image credits: Yutaka Ehara

**Figure 3:**
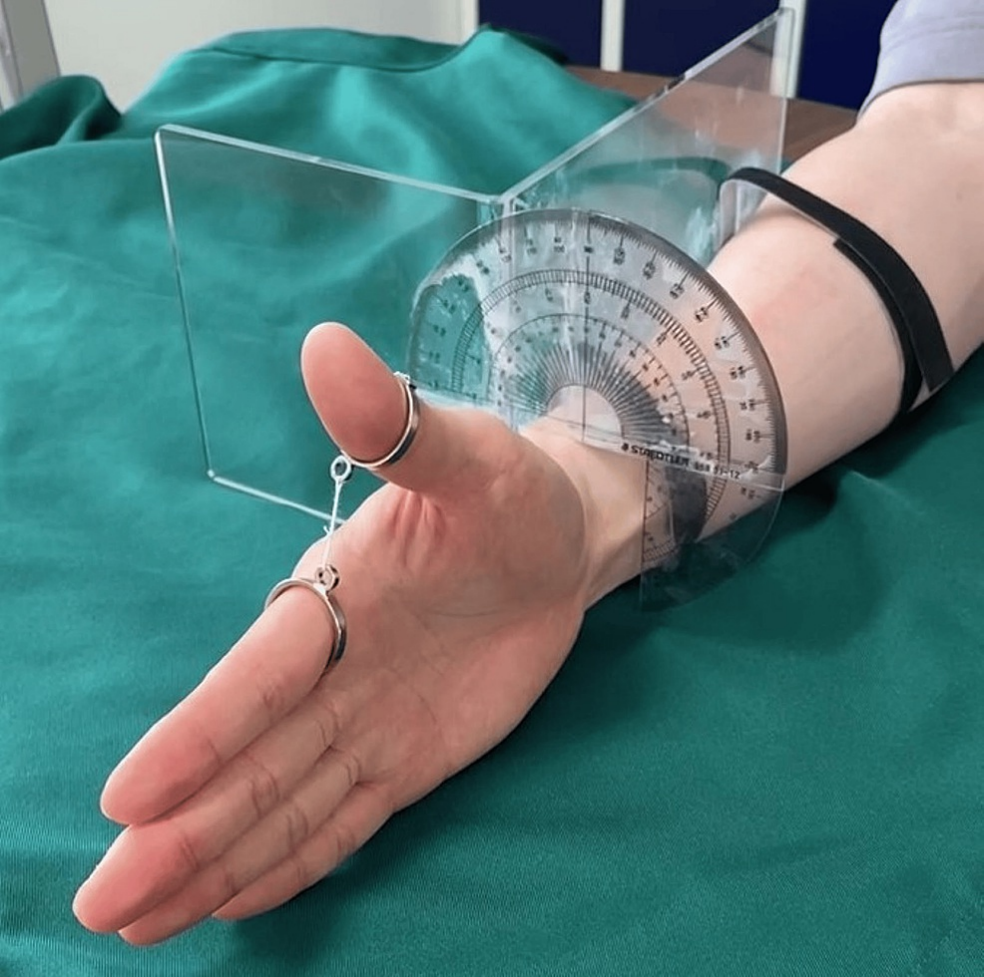
The angular device for measurement The distance (r) = 3 cm between the interphalangeal (IP) joint of the thumb and the metacarpophalangeal (MP) joint of the index finger was kept, and the protractor was placed vertically

Based on previous reports [16,17], the abduction motion of the thumb was simulated as the motion of a cone with the CM joint at its apex. The abduction motion of the thumb was defined as a planar rotational motion of the thumb IP joint, with the second MP joint as the rotation center and the second metacarpal as the base axis. The movement of the thumb was represented in a radian system and the position of the thumb IP joint was defined as the rotational displacement coordinates (r, θ) with the rotational angle θ set at 0° on the palmar plane and the distance r from the base axis. As per this definition, the position of the thumb’s IP joint was determined (Figure 2). To reproduce the motion of the thumb under these conditions, we created the device to keep the distance (r) between the IP joint of the thumb and the MP joint of the index finger at 3 cm (Figure 3). Evaluator A was an orthopedic surgeon with eight years of clinical experience, and evaluator B was a physical therapist with 10 years of clinical experience. Participants were instructed to assume a sitting position, with the elbow joint in mild flexion, mid-forearm rotation, and mid-wrist position on a desk in front of the body. Measurements were taken using the angular device described above. The rotation angle was measured in ten angles of 10° each, between 0° and 90°, from the palmar plane.

3. Data acquisition and image processing using MediaPipe

**Figure 4:**
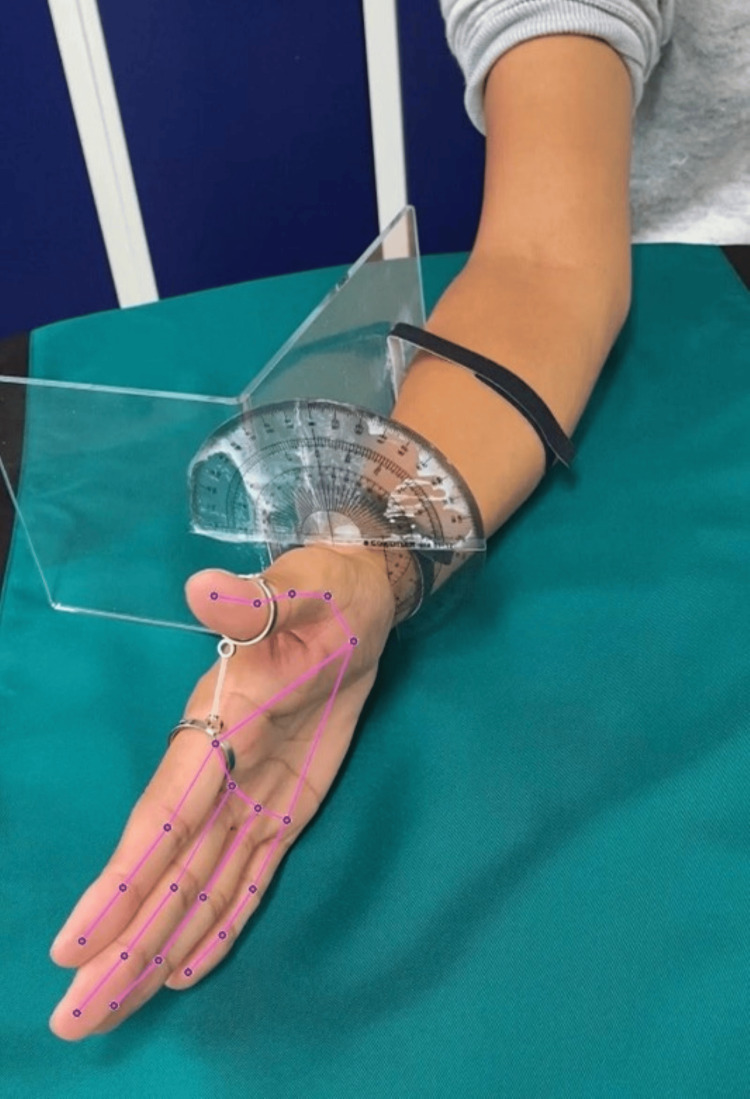
Example of hand coordinates detected by MediaPipe

**Figure 5:**
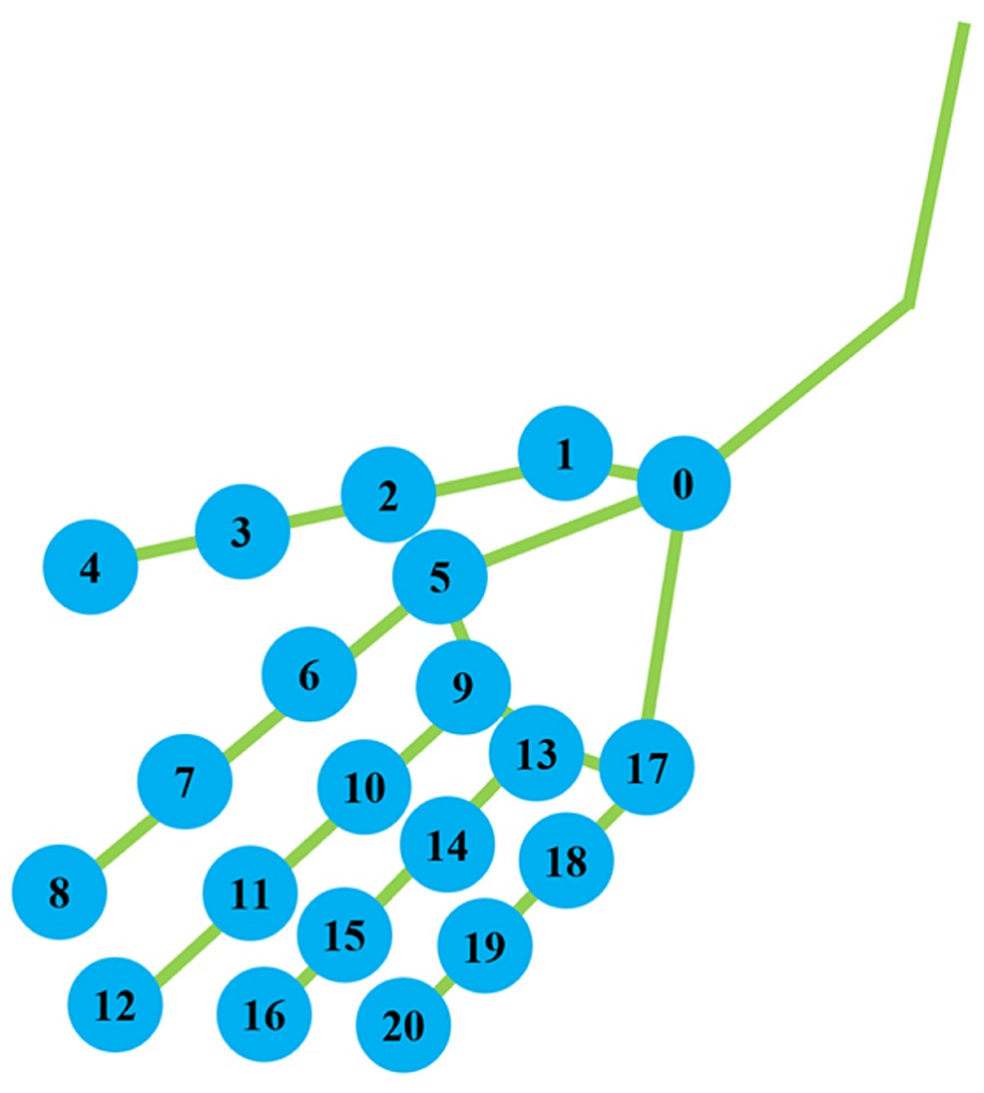
MediaPipe landmarks: detecting the bounding box of a relatively rigid body part of a hand and predicting the hand skeleton (0) WRIST. (1) THUMB_CMC. (2) THUMB_MCP. (3) THUMB_IP. (4) THUMB_TIP. (5) INDEX_FINGER_MCP. (6) INDEX_FINGER_PIP. (7) INDEX_FINGER_DIP. (8) INDEX_FINGER_TIP. (9) MIDDLE_FINGER_MCP. (10) MIDDLE_FINGER_PIP. (11) MIDDLE_FINGER_DIP. (12) MIDDLE_FINGER_TIP. (13) RING_FINGER_MCP. (14) RING_FINGER_PIP. (15) RING_FIGER_DIP. (16) RING_FIGER_TIP. (17) PINKY_MCP. (18) PINKY_PIP. (19) PINKY_DIP. (20) PINKY_TIP

Videos were captured for 1 s. The video files were processed using the Python library of MediaPipe to obtain hand coordinates. Figure 4 shows an example of an image analyzed using MediaPipe [18]. From the x and y coordinates computed by MediaPipe, eight parameters were calculated, including the distance, angle, and size of the thumb and index finger, as well as the size of the palm. These parameters were then used as a dataset for ML purposes. The methods for calculating the parameters are summarized in Table 1. A diagram of the points used for each parameter is shown in Figure 5. The parameters utilized for analyzing the rotational movement of the thumb are presented in Table 1.

**Table 1:** Various parameters used for the training of machine learning models true_angle: the value of the rotation angle measured by the protractor. tip_angle: angle formed by points (4), (1), and (5). mp_angle: angle formed by points (2), (1), and (5). palm_angle: the angle formed by points (5), (1), and (17). norm_tip_distance: the value of the distance from (5) to (4) divided by the distance from (5) to (8). norm_ip_distance: the value of the distance from (5) to (3) divided by the distance from (5) to (8). norm_mp_distance: the value of the distance from (5) to (2) divided by the distance from (5) to (8). norm_tip_size: the value of a cross-product of the vector from (1) to (4) and the vector from (1) to (5) divided by the square of the distance from (5) to (8). norm_mp_size: the value of a cross-product of the vector from (1) to (2) and the vector from (1) to (5) divided by the square of the distance from (5) to (8). norm_palm_size: the value of a cross-product of the vector from (1) to (5) and the vector from (1) to (17) divided by the square of the distance from (5) to (8)

Parameter	Definition	Explanation
true_angle	Value on protractor	True value
tip_angle	∠(4)-(1)-(5)	Thumb rotation angle
mp_angle	∠(2)-(1)-(5)	Thumb rotation angle
palm_angle	∠(5)-(1)-(17)	Palmar angle
norm_tip_distance	(5)-(4) /(5)-(8)	Normalized distance from the base axis to the tip of the thumb
norm_ip_distance	(5)-(3) /(5)-(8)	Normalized distance from the base axis to the IP joint of the thumb
norm_mp_distance	(5)-(2) /(5)-(8)	Normalized distance from the base axis to the MP joint of the thumb
norm_tip_size	(1)-(4) × (1)-(5) /(5)-(8)^2^	Area of the thumb
norm_mp_size	(1)-(2) × (1)-(5) /(5)-(8)^2^	Area of the thenar eminence
norm_palm_size	(1)-(5) × (1)-(17) /(5)-(8)^2^	Area of the palm

4. Machine learning (ML)

**Figure 6:**
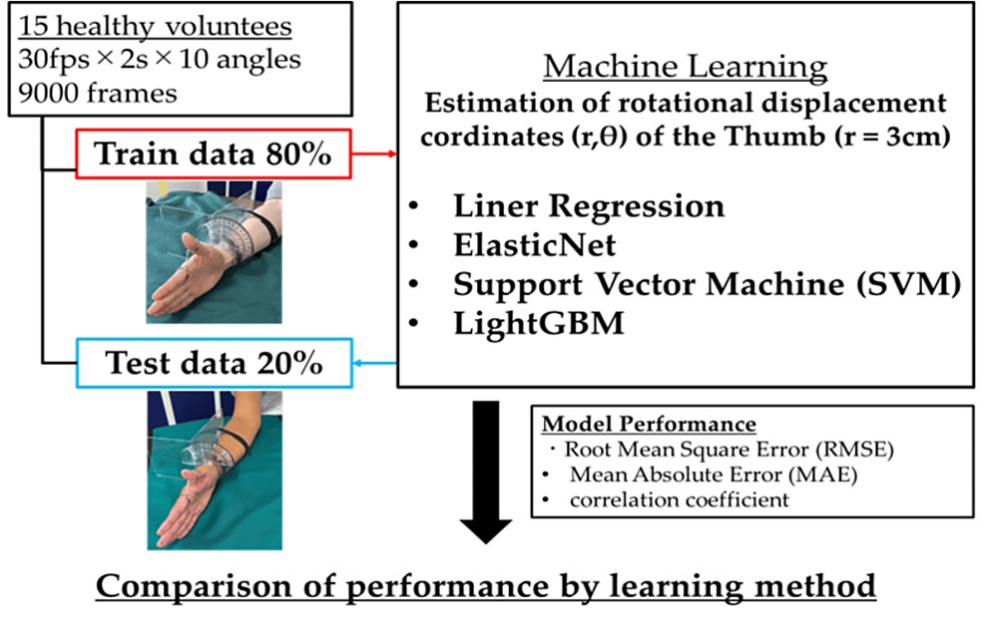
Workflow of the data acquisition and machine learning A total of 9000 images were captured at 30 fps over 2 s from 15 participants with 10 different thumb angles ranging from 0 to 90°. The distance (r) = 3 cm between the IP joint of the thumb and the MP joint of the index finger was maintained. The captured images were randomly separated, allocating 80% as training data to fine-tune the model’s hyperparameters and 20% as test data to evaluate the efficacy of each ML model. To identify the best hyperparameters for each ML algorithm with the help of the training data, we used the root mean square error (RMSE), the mean absolute error (MAE), and the correlation coefficient as the key indicators to estimate and compare the precision of the models utilized

We compared the performance of four ML algorithms: linear regression, ElasticNet, SVM, and LightGBM [21]. The algorithms estimate the rotation angle of the thumb (θ) using parameters calculated from the estimated joint coordinates. Traditional linear regression and ElasticNet were employed as a basic regression technique. SVM is an algorithm that performs classification or regression by determining a boundary line or hyperplane that separates two classes of data, whereas the LightGBM framework enhances processing speed, minimizes memory consumption, and offers superior categorization capabilities while avoiding the issue of overfitting. For model training purposes, Python-based Scikit-learn, a library dedicated to ML, was utilized. The workflow of this experiment is shown in Figure 6. In this way, the accuracy of estimating the rotation angle of the thumb was calculated. In total, 9000 images were used from 15 participants with 10 different thumb angles ranging from 0 to 90. The collected images were randomly divided, allocating 80% as training data to fine-tune the model’s hyperparameters and 20% as test data to assess the efficacy of each ML model.

**Figure 7:**
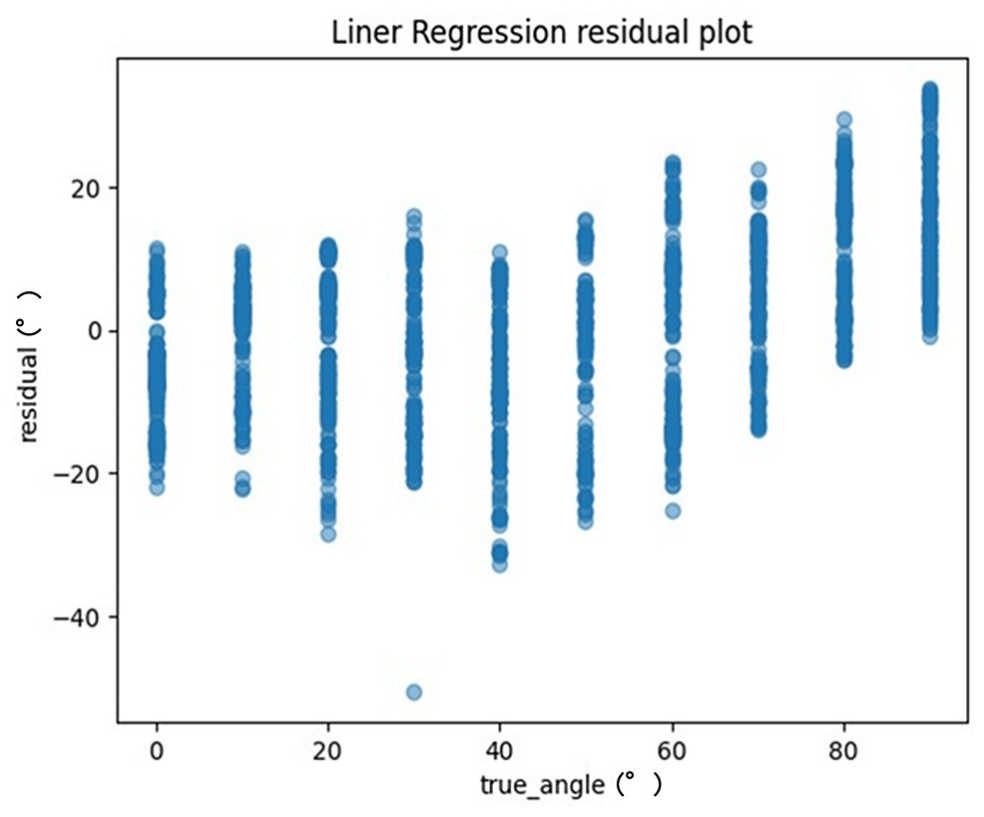
The residuals (actual angles–predicted angles) of the linear regression model were plotted and compared against the actual angles for the test data

**Figure 8:**
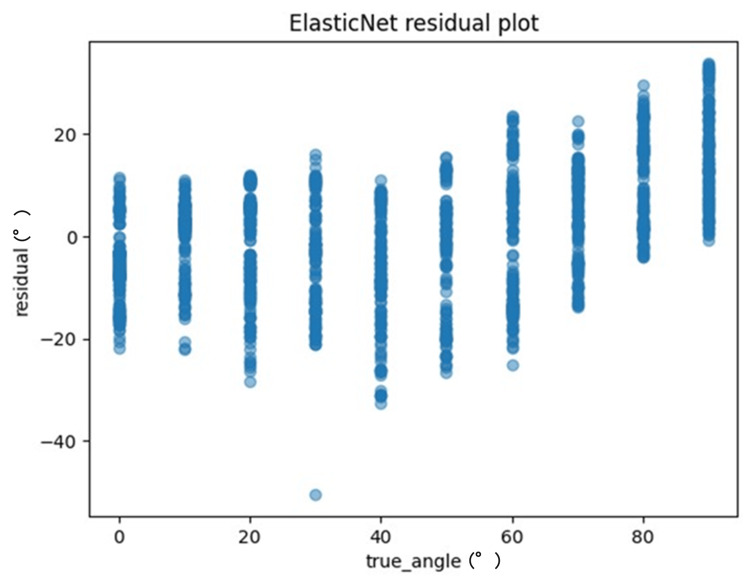
The residuals of the ElasticNet model were charted and evaluated against the actual angles for the test data

**Figure 9:**
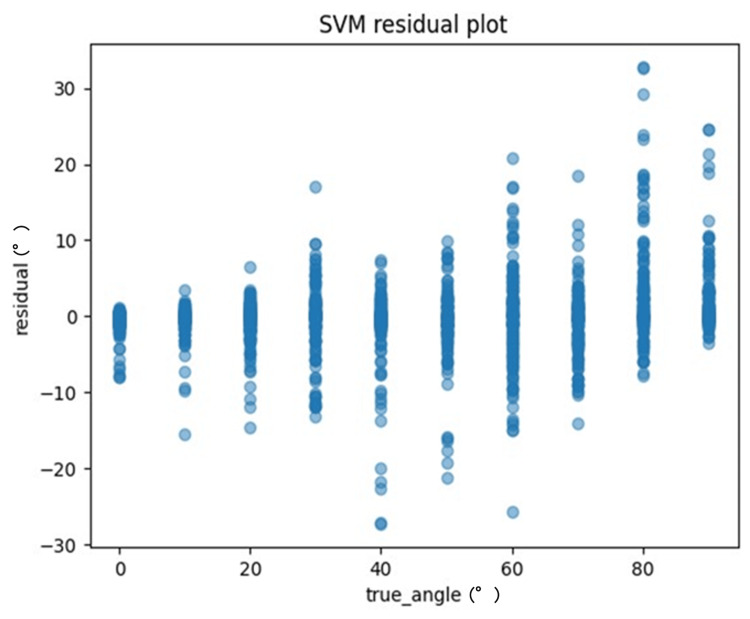
The residuals of the support vector machine (SVM) model were graphed and compared against the actual angles for the test data

**Figure 10:**
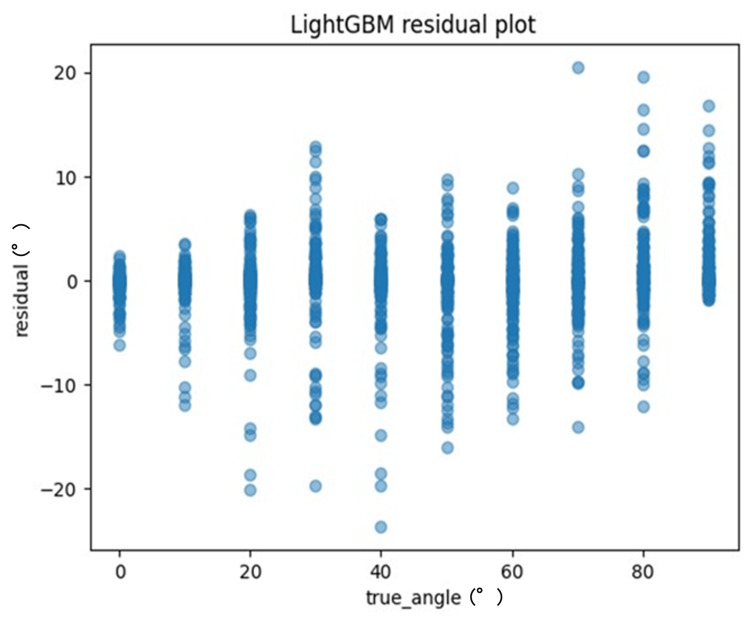
The residuals of the LightGBM model were graphically represented and juxtaposed with the actual angles for the test data

One method to evaluate the quality of regression analysis involves the residual plot. The residual plot displays the differences (residuals) between predicted and actual values in regression analysis. For each ML model, the actual values (true angle) were plotted on the x-axis and the residuals (actual angles-predicted angles) on the y-axis (Figures 7-10). Residuals close to zero were indicative of the model adequately capturing the data. Upon identifying the best hyperparameters for each ML algorithm with the help of the training data, we chose the RMSE, the MAE, and the correlation coefficient as the key indicators to evaluate and compare the precision of the models utilized.

In the model with a higher accuracy, feature importance and SHapley Additive exPlanations (SHAP) values were used to visualize the parameters crucial for estimating the rotation angle of the thumb. The features were normalized by the sum of all feature values present in the tree. By dividing by the total number of trees in the ML, the overall importance of the features was obtained. Furthermore, the contribution of each feature to the prediction was assessed using the SHAP values. Based on the game theory, SHAP values were defined as each feature’s contribution to the model’s predictions. SHAP values are useful for enhancing the interpretability of a model and are particularly beneficial in complex models [22]. Every analysis of the ML models was conducted with the Scikit-learn v1.0.2 library within the Python v3.8 setup.

## Results

**Figure 11:**
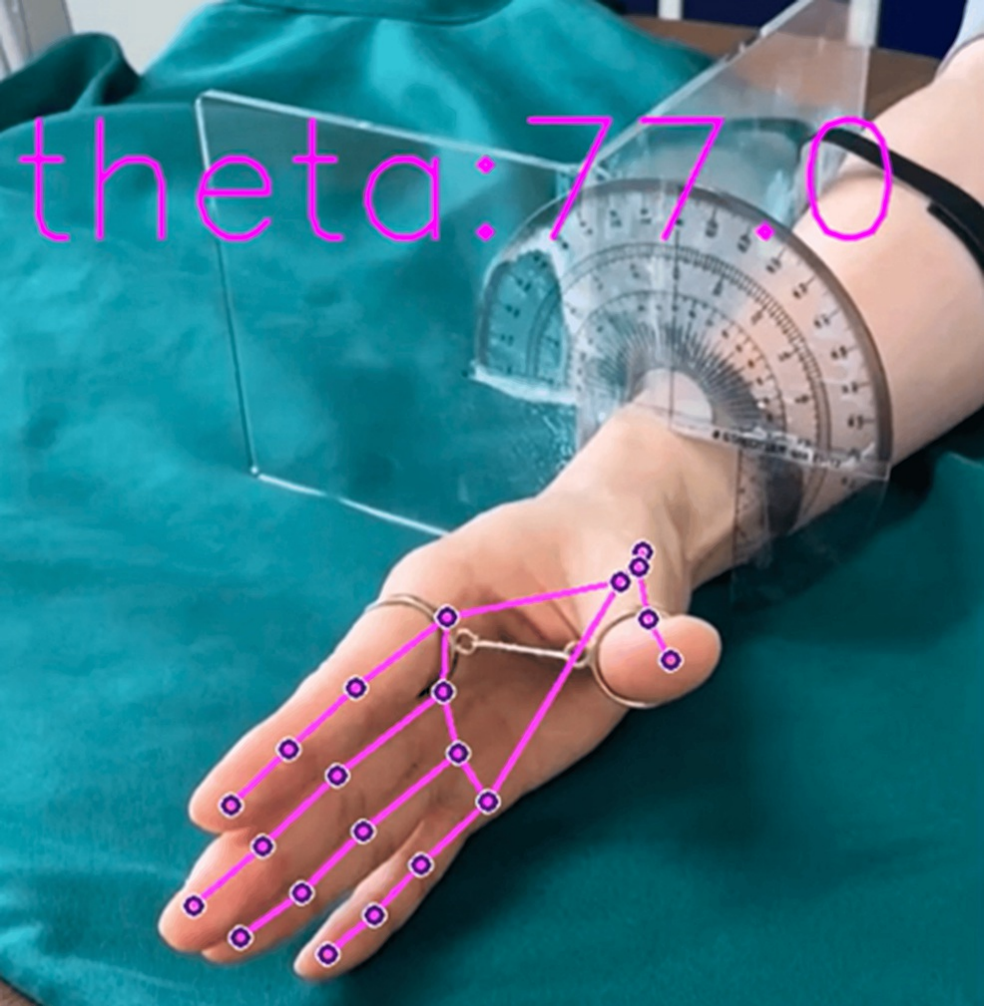
Example of hand coordinate detection and estimated rotation angle (θ: theta) by the machine learning model

**Figure 12:**
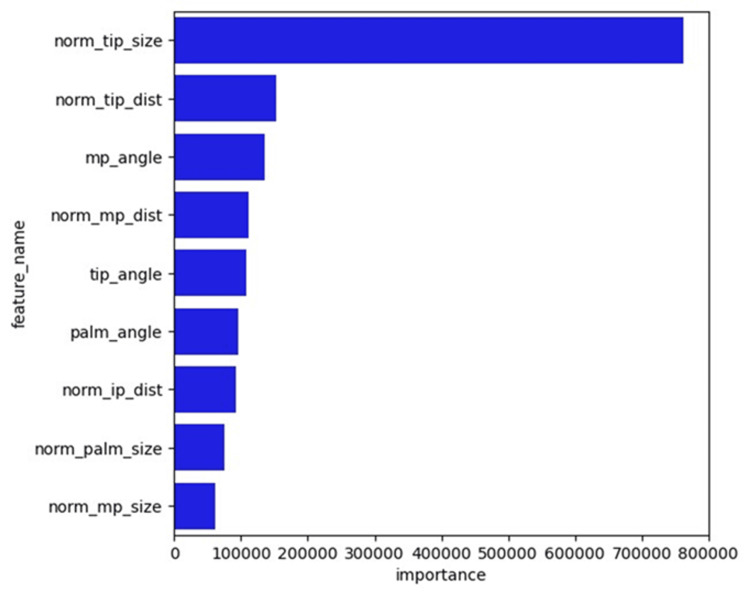
The feature importance of the LightGBM model Top three essential features: norm_tip_size, norm_tip_dist, and mp_angle

The recordings were processed as 9000 images, and the model was trained using ML models (Figures 6-9, 11-12). The results are shown in Table 2. Compared to these ML models, LightGBM had the highest accuracy. The mean and standard deviation of each residual of the ML model for each angle are summarized in Tables 3-6. The mean residual of the SVM and LightGBM were smaller than that of linear regression and ElasticNet. The smallest residual value was observed at a rotation angle of 60. An example of hand coordinate detection and rotation angle (θ) estimated by this ML model is shown in Figure 11.

**Table 2:** The accuracy of each machine learning model RMSE: root mean square error; MAE: mean absolute error; SVM: support vector machine

	Linear regression	ElasticNet	SVM	LightGBM
RMSE (°)	12.23	12.23	4.70	4.58
MAE (°)	9.95	9.95	2.56	2.62
Correlation coefficient	0.91	0.91	0.99	0.99

**Table 3:** The mean and standard deviation of each residual for each angle of the linear regression model

Linear regression	Residual	Residual
	Mean (°)	Standard deviation (°)
True_Angle (°)		
0	−6.05	7.24
10	−0.15	7.15
20	−1.55	10.17
30	−4.71	11.0
40	−7.09	11.28
50	−3.52	12.02
60	−1.19	13.06
70	4.29	9.17
80	11.31	9.42
90	15.86	9.6

**Table 4:** The mean and standard deviation of each residual for each angle of the ElasticNet model

ElasticNet	Residual	Residual
	Mean (°)	Standard deviation (°)
True_Angle (°)		
0	−6.06	7.23
10	−0.15	7.15
20	−1.55	10.17
30	−4.71	11.04
40	−7.09	11.28
50	−3.53	12.0
60	−1.19	13.05
70	4.29	9.17
80	11.31	9.42
90	15.86	9.64

**Table 5:** The mean and standard deviation of each residual for each angle of the SVM model SVM: support vector machine

SVM	Residual	Residual
	Mean (°)	Standard deviation (°)
True_Angle (°)		
0	−0.49	1.36
10	−0.49	1.9
20	−0.53	2.48
30	−0.39	4.59
40	−1.27	5.20
50	−0.86	4.84
60	−0.13	6.36
70	−0.78	4.36
80	3.17	7.22
90	2.33	4.77

**Table 6:** The mean and standard deviation of each residual for each angle of the LightGBM model

LightGBM	Residual	Residual
	Mean (°)	Standard deviation (°)
True_Angle (°)		
0	−0.49	1.38
10	−0.49	1.95
20	−0.53	2.48
30	−0.39	4.59
7.22	−1.27	5.20
4.77	−0.86	4.84
60	−0.13	6.37
70	−0.78	4.36
80	3.17	7.22
90	2.33	4.77

**Figure 13:**
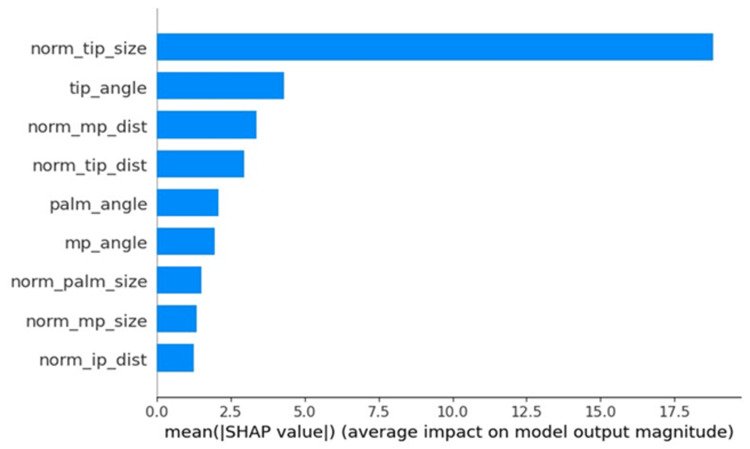
SHAP value of the LightGBM model The top three essential features: norm_tip_size, tip_angle, and norm_mp_dist SHAP: SHapley Additive exPlanation

The parameters norm_tip_size, norm_tip_dist, and mp_angle had higher scores when the estimating angles were evaluated using the feature importance (Figure 12), which shows that the feature has a bigger effect on the model that is being used to predict the rotation angle (θ) of the thumb. The parameters norm_tip_size, tip_angle, and norm_mp_dist were higher for SHAP values (Figure 13), indicating the magnitude of the feature contribution. Both identified that norm_tip_size, calculated as the area of the parallelogram formed by the thumb, and the second metacarpal bone, normalized by the squared distance of the fingers, showed the highest correlation with the thumb rotation angle.

**Figure 14:**
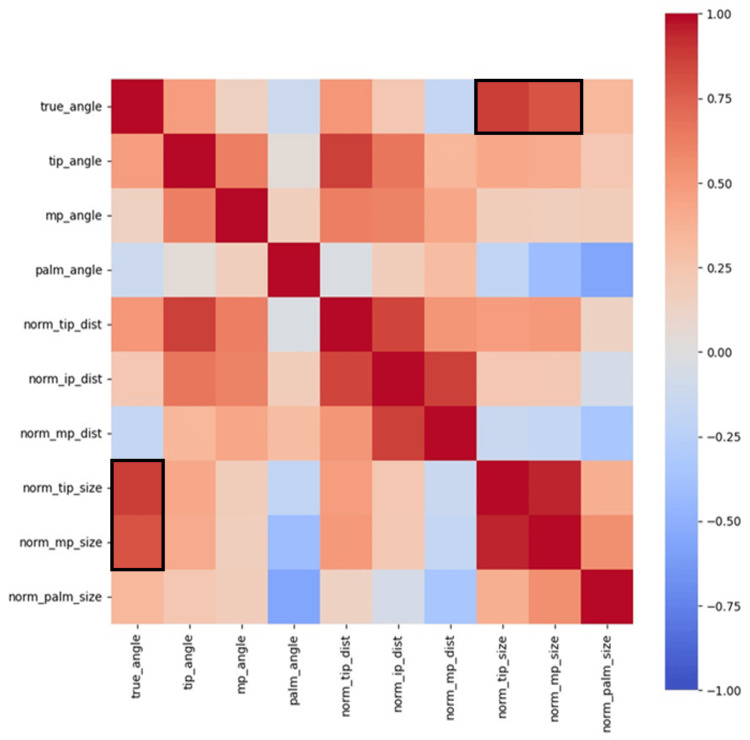
Heatmap of each parameter Warm colors indicate a positive correlation, while cool colors signify a negative correlation. The true_angle demonstrates a strong positive correlation with norm_tip_size and norm_mp_size

As part of the exploratory data analysis (EDA), a heatmap showcasing the correlation between the parameters is shown in Figure 14. According to the heatmap, the true angle is positively correlated with norm_tip_size and norm_mp_size. The heatmap results are consistent with feature importance and the SHAP value results.

## Discussion

Hands perform complex movements, including pinching and gripping, and the thumb plays a crucial role in the functionality of the hand. Thumb movements are typically described as palmar abduction, radial abduction, adduction, palmar adduction, and opposition. Traditionally, these movements have been measured using a goniometer. Originally, abduction motion was divided into palmar abduction and radial abduction. However, if the CM, MP, and IP joints are on the same line, it can be easily expressed by approximating them as a cone. Kuo et al. [23,24] acknowledged that the head of the first metacarpal bone performs spherical movements centered on the CM joint. In their study, the forearm was immobilized, and an electromagnetic sensor was attached to the dorsal side of the thumb MP joint to measure the range of motion of the metacarpal head. This approach allowed them to consolidate the complex movements of the thumb into a single three-dimensional display [23,24]. These 3D measurement models require a complex setup of measurement devices. In the present study, we used a pose estimation AI combined with an ML model assuming the thumb rotation motion as a cone motion. To accommodate such movement of the thumb, we defined the thumb IP joint rotating around the second metacarpal bone, using the second MP joint as the rotational center and maintaining a constant distance as the radius. As shown in Figure 2, r was set to 3 cm and θ was estimated by the ML model. Using MediaPipe, hand coordinates were detected, and training parameters were created. ML was then applied to these parameters to estimate the angle θ without using an angular clock.

**Figure 15:**
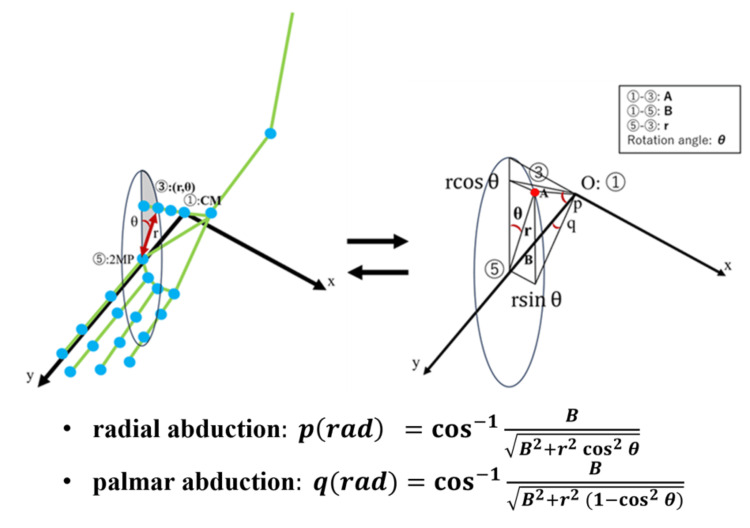
Conversion from rotational displacement coordinates (r, θ) to radial abduction and palmar abduction

The movement of the thumb could also be represented in a polar coordinate system. In a normal joint, the axis of rotation for flexion-extension is in the trapezium, and that for abduction-adduction is in the base of the thumb metacarpal [17]. The coordinate system was defined as follows: the origine (O) indicates the CM joint; the x- and y-coordinates represent the horizontal and vertical coordinates from the detected palm center, respectively; and the z-coordinate indicates the direction parallel to the palmar surface in the neutral position of pronation and supination (the direction of gravity). Assume that the distance from the CM joint to the IP joint is A, and the distance from the CM joint to the 2MP joint is B, and the polar coordinates of the thumb IP joint are expressed as (r𝐬𝐢𝐧𝜽, B, r 𝐜𝐨𝐬𝜽 ). Radial deviation and palmar deviation can be calculated from the rotational displacement coordinates (r, θ) using the following formula depicted in Figure 15. Using this method, radial abduction and palmar abduction can be evaluated simultaneously.

In recent years, AI and ML have been used in the field of orthopedics [25]. For instance, it has been reported that the prediction of factors helps improve the prognosis of ACL reconstruction using ML models such as ElasticNet and SVM [26]. Villalba-Meneses et al. described the classification using ML models such as SVM of motion capture data obtained from the range of motion exercises among healthy and clinically diagnosed patients with low back pain [27]. Kusunose et al. indicated that the integration of MediaPipe with ML models using liner regression and LightGBM for posture analysis has improved accuracy in assessing shoulder abduction angles [20]. In this study, four ML algorithms - linear regression, ElasticNet, SVM, and LightGBM - were applied and the LightGBM model demonstrated a high accuracy, as the change in each parameter relative to the true value of each parameter indicated a nonlinear change. The large amount of variation in the data suggests that LightGBM, a nonlinear model, achieved better results than linear regression, ElasticNet, and SVM, which are linear models.

The study found a high correlation between the posture estimation with AI and the actual movement angles of the thumb. This level of accuracy was favorable even compared to previous research. Gu et al. have reported that the average difference from the goniometer was −2.21 ± 9.29° when analyzing images of a specific protocol position captured with a smartphone using only MediaPipe [4]. Therefore, we combined MediaPipe and ML algorithms. In this study, the average difference from the goniometer was 0.056 ± 1.39° with a smartphone using MediaPipe and LightGBM. Integrating MediaPipe and ML improved the accuracy of the estimation of the thumb rotation angle.

In the field of AI, the explainability of ML models is important. Prior research has incorporated feature importance and SHAP analyses to ensure transparency and interpretability [20,22]. In the present study, the feature values that were important for predicting the rotation angle (θ) were also assessed utilizing feature importance and the SHAP values. Both highlighted that norm_tip_size, calculated by normalizing the area of the parallelogram formed by the thumb and the second metacarpal bone, divided by the squared distance of the index finger, showed the strongest correlation with the thumb rotation angle. Since norm_tip_size is a normalized value regardless of the distance between the camera and the hand, it might be useful in various camera settings.

The estimation model developed in this research can display angles in real time without using complicated devices, which is a characteristic not reported in previous studies and considered innovative. If a tablet device is available, it enables remote, real-time consultations and measurements, expanding the potential for remote medical and rehabilitation applications. The possibility of using smartphones from various positions for analyzing the movement of the thumb and other fingers holds promise for enhancing efficiency in motion analysis and contributing to applications in remote healthcare and rehabilitation.

There are several limitations to this study, primarily the potential for inaccuracy of the protractor used as the true value measurement. Rigorous training sessions for evaluators and clear instructions for participants are essential to implement to minimize measurement variability. It would be more accurate to use an infrared camera and marker as the gold standard. However, given the complexity of setting up the instruments and their application to telemedicine in the future, we used a simple experimental system with a web camera and an angular system device. The second limitation pertains to the fact that the distance from the IP joint to the second MP joint does not remain constant in the rotation movement of the thumb in reality. The third relates to the chance that the coordinates estimated by MediaPipe from the surface may not align with the actual joint coordinates. The fourth limitation is the small number of cases that serve as our source, thus the risk of overfitting cannot be ruled out. Finally, this study involved healthy volunteers and limited positions. This study is preliminary, and more studies involving actual patients are strongly recommended. Further research is required to understand the correlation between these metrics and the functional aspects of the thumb.

## Conclusions

In this study, we demonstrated the potential of estimating the thumb rotation angle using MediaPipe combined with the LightGBM model. The rotation angle was estimated by four ML models - linear regression, ElasticNet, SVM, and LightGBM - by using parameters such as angles, distances, and areas calculated from hand coordinates detected by MediaPipe. The models were evaluated using the RMSE, the MAE, and the correlation coefficient. The LightGBM model achieved high values for RMSE, MAE, and correlation coefficient. Both feature importance and the SHAP values identified that norm_tip_size (area of the thumb: value of a cross-product of the vector from THUMB_CMC to THUMB_TIP and the vector from THUMB_CMC to INDEX_FINGER_MCP divided by the square of the distance from INDEX_FINGER_MCP to INDEX_FINGER_TIP) showed the highest correlation with the thumb rotation angle.

The estimation model developed in this study enabled the display of angles in real-time with high accuracy without the need for complex devices. This characteristic has not been reported in previous methods and is considered innovative. The availability of tablet devices enables remote, real-time consultations and measurements, opening up new possibilities for remote medical and rehabilitation applications. To sum up, by utilizing a cone model of the thumb from videos captured from the palmar side with a smartphone and combining MediaPipe with ML, it is possible to estimate the rotation angle of the thumb with high accuracy.
